# The concept of radiation-enhanced stem cell differentiation

**DOI:** 10.1515/raon-2015-0022

**Published:** 2015-08-21

**Authors:** Adam A. Mieloch, Wiktoria M. Suchorska

**Affiliations:** Radiobiology Laboratory, Department of Medical Physics, The Greater Poland Cancer Centre

**Keywords:** stem cells, ionizing radiation, differentiation, regenerative medicine, tissue engineering

## Abstract

**Background:**

Efficient stem cell differentiation is considered to be the holy grail of regenerative medicine. Pursuing the most productive method of directed differentiation has been the subject of numerous studies, resulting in the development of many effective protocols. However, the necessity for further improvement in differentiation efficiency remains. This review contains a description of molecular processes underlying the response of stem cells to ionizing radiation, indicating its potential application in differentiation procedures. In the first part, the radiation-induced damage response in various types of stem cells is described. Second, the role of the p53 protein in embryonic and adult stem cells is highlighted. Last, the hypothesis on the mitochondrial involvement in stem cell development including its response to ionizing radiation is presented.

**Conclusions:**

In summary, despite the many threats of ionizing radiation concerning genomic instability, subjecting cells to the appropriate dosage of ionizing radiation may become a useful method for enhancing directed differentiation in certain stem cell types.

## Introduction

Stem cells (SCs) possess a set of unique advantages, including the ability to replicate and the ability to differentiate into many different types of cells, called “pluripotency”. Due to the pluripotent characteristic of these cells, they play a pivotal role in tissue development and maintenance by replenishing the depletion of cells caused by damaging factors or that occurs physiologically during tissue turn-over.^1^ The majority of recent studies have mainly focused on two types of stem cells: Embryonic stem cells (ESCs) and adult stem cells (ASCs), also known as somatic tissue or mesenchymal stem cells.^2^ ESCs are derived from the inner cell mass of the blastocyst and are capable of differentiating into the three embryonic germ layers: ectoderm, mesoderm and endoderm, thus contributing to the formation of almost every cell type. ASCs reside in tissue-specific niches in a quiescent state. Upon activation, they undergo asymmetric division, which simultaneously increases the number of cells in the niche and the number differentiating into tissue specific lineages, providing cells required for tissue regeneration.^3^,^4^

Stem cells have had a significant impact on the progress of many fields of biotechnology, including cell-based regenerative therapies, drug testing and screening, disease modeling, side effects in radiotherapy and many more. In 2006, Yamanaka *et al*. announced a breakthrough finding in regenerative medicine, describing the reprogramming of mouse adult fibroblasts into induced pluripotent stem cells (iPSs) by introducing four factors: Oct3/4, Sox2, c-Myc, and Klf4. iPSs in many aspects resemble ESCs.^5^ This discovery solved many of the ethical disputes concerning the procurement of ESCs from human embryos and began a new era in regenerative medicine.

Since then, many attempts to harness the pluripotency of stem cells into directed differentiation have been successful.^6^,^7^ Some of developed protocols require the formation of embryoid bodies (EBs) prior to further differentiation. EBs are three-dimensional cellular aggregates obtained by spontaneous differentiation of ESCs or IPSs. EBs consist of ESCs that are mostly differentiated into the embryonic germ layers: ectoderm, endoderm and mesoderm.^8^ The differentiation process in many aspects mimics early mammalian embryogenesis, including cell to cell interactions. Moreover, the most essential method of EBs formation is based on suspension culture deprived of antidifferentiation factors. Due to simple methodology and similarity to embryogenesis, EBs are widely utilized as an intermediate stage during *in vitro* differentiation of both human and murine ESCs. 9

Currently, many stem cell-based therapies are undergoing clinical trials, for example, “Intravenous Stem Cells After Ischemic Stroke”, “Human Neural Stem Cell Transplantation in Amyotrophic Lateral Sclerosis (ALS)” and “Treatment of Knee Osteoarthritis by Intra-articular Injection of Bone Marrow Mesenchymal Stem Cells”.^10^–^12^ These trials are just a few from a still enlarging group of studies investigating the potential applications of stem cells in regenerative medicine, which indicates a growing need for reliable methods of directed differentiation of SCs.

Ionizing radiation (IR) has been used for many years as a basic tool in cancer treatment.^13^ The response of non-stem cells to irradiation has been extensively investigated by a number of studies, and to date, many molecular mechanisms of this phenomena have been thoroughly elucidated.^14^–^16^ However, based on the current understanding concerning non-SCs, the radioresponse of SCs cannot be anticipated and it could result in unexpected outcomes.

Radiation-induced differentiation has already been reported in multiple studies^17^,^18^; however, it has not been investigated as a potential tool in stem cell differentiation protocols. The main goal of this review is to present research based indications that radiation-enhanced differentiation is a promising technology for further development of stem cell engineering.

### Radiation-induced DNA damage response in stem cells

Radiation-induced damage to genomic DNA triggers a cascade of biochemical reactions known as the DNA damage response (DDR), which includes cell cycle arrest, DNA repair and, in the case of unmanageable lesions, senescence or apoptosis. The functional mechanism of DNA damage repair is crucial for the maintenance of genomic stability.

The most dangerous type of DNA lesions are double strand breaks (DSBs), which are usually caused by IR or free radical exposure. Repair of DSBs is driven by two major pathways: homologous recombination (HR) and non-homologous end joining (NHEJ).^19^ In the process of HR, sister chromatids serve as a template; thus, the repair is considered error-free. NHEJ does not utilize sister chromatids as a template and is therefore significantly more prone to error introduction. Depending on the phase of the cell cycle, one of the pathways is used predominantly. The requirement for sister chromatids in HR restrains its activity to the S and G2 phases. The NHEJ response dominates through the rest of the cell cycle.^20^ There are also other types of DNA damage repair mechanisms: nucleotide excision repair (NER), base excision repair (BER) and mismatch repair (MMR). However, their contribution to radiation-enhanced differentiation seems to be negligible and will not be considered in this study.

#### DNA damage repair in embryonic stem cells (ESCs)

It has been proven that the mechanisms of DNA damage repair in ESCs are more efficient compared to other cell types.^21^ ESCs display a unique cell cycle structure. The G1 phase is significantly shortened and the G1 to S transition is facilitated in order to promote rapid self-renewal. Consequently, the majority of the ESC population is in the phases of cell cycle where sister chromatids are available for use as a template. Due to this phenomena, ESCs predominantly utilize high-fidelity HR.^22^

ESCs serve as a pool of cells for the development of the whole organism. Therefore, DNA repair in these cells requires high efficiency and accuracy in order to provide genomic stability. In the case of insults in the genomic DNA that cannot be repaired, the cell undergoes apoptosis, which is significantly facilitated by a mechanism known as mitochondrial priming in ESCs.^23^ Mitochondrial priming is determined by the equilibrium between levels of anti-apoptotic and pro-apoptotic proteins of the B-cell lymphoma 2 (Bcl-2) protein family. ESCs display elevated levels of pro-apoptotic proteins within the mitochondria. Consequently, the initiation of apoptosis requires a considerably weaker stimuli in order to cross the apoptotic threshold. This phenomenon ensures elimination of genetically unstable cells and prevents further transmission of mutations.

A previous study by Sokolov and Naumann revealed that human embryonic stem cells (hESCs) undergo apoptosis after relatively low-dose irradiation. In the study, a 1.0 Gy dose of X-ray radiation triggered robust apoptosis. Conversely, doses of 0.5 Gy and 0.2 Gy did not increase the apoptotic response.^24^ A study by Lan *et al.* reported that a 2.0 Gy dose of X-ray radiation resulted in an almost 60% decrease in the survival rate of hESCs 5 days post-irradiation. The same study found that X-ray irradiation elevated metabolic activity (XTT assay) 1.5-fold after a 2.0 Gy dose and 2.5-fold after a 5.0 Gy dose. The same dosage of 2.0 Gy and 5.0 Gy resulted in elevated levels of reactive oxygen species (ROS) and nitrogen (RNS) species for 1 week following exposure.^25^

#### DNA damage repair in adult stem cells (ASCs)

ASC sensitivity to irradiation varies greatly, depending on their type and developmental stage. However, it is postulated that the DNA repair mechanism becomes less efficient upon differentiation in general. Therefore, ASCs display reduced DNA damage repair (DDR) capabilities in comparison to ESCs, which has been shown previously.^26^ It is important to note that the mechanism of DDR in ASCs is distinctly different than the one observed in ESCs.^27^ ASCs reside in a quiescent state in the G0 phase of the cell cycle. Slower cell cycle progression corresponds to a higher radioresistance.^28^,^29^ Therefore, despite a lower efficacy of DDR, ASCs exhibit a lower sensitivity to IR compared to the rapidly dividing ESCs. It has been shown that upon DNA damage, ASCs can exit quiescence and progress into the G1 phase, in which error-prone NHEJ repair is performed.^30^ Consequently, ASCs are more susceptible to DNA damage accumulation, which can be passed onto progeny.

In 1996, Schwenke *et al.* 17 found that γ-irradiation of murine erythroid progenitor cells resulted in enhanced differentiation. This observed enhancement was determined to be due to the omission of mitotic cell cycling, which is necessary for progenitor cells to undergo terminal differentiation. Moreover, Zheng *et al.* 31 found that DSB suppresses the self-renewal and promotes the further differentiation of neuronal stem cells (NSCs) in a p53-dependent manner.

### Role of p53 in stem cells

The p53 protein has been widely studied for many years, and a number of its properties have been elucidated.^32^ However, the complexity of its interactions and associations with various molecular processes has left many novel functions of this protein remaining to be discovered.

p53 is a tumor suppressor protein responsible for the induction of reversible cell cycle arrest, which enables DNA repairs to be conducted, and the initiation of apoptosis in the case of irreversible DNA damage. p53 is a transcription factor that, upon activation, binds to the promoters of target genes, either inducing or repressing their transcription depending on the gene.^33^ p53 can trigger apoptosis via two pathways: the transcriptional (intrinsic) pathway, as described above, or the *non*-transcriptional (mitochondrial) pathway by direct interactions with *pro*- and *anti*-apoptotic proteins. The main target genes for its proapoptotic activity include p53 upregulated modulator of apoptosis (Puma) and Bcl-2-associated X (Bax) proteins, which belong to the Bcl-2 family.^34^ DNA damage results in ataxia telangiectasia mutated (ATM) protein activation, which drives mouse double minute 2 homolog (Mdm2) polyubiquitination and further degradation. Mdm2 is an oncoprotein that mediates p53 polyubiquitination and further degradation by the 26S proteasome. Therefore, Mdm2 degradation contributes to the increased stability of p53. It is worth noting that other mechanisms of p53 regulation also exist. Furthermore, p53 performs a regulatory function over cell proliferation by controlling the expression of the p21 protein, known as cyclin-dependent kinase inhibitor.^35^ Silencing of p53 expression has also been shown to increase the efficiency of reprogramming in iPSs generation, indicating its contribution to the maintenance of a differentiated state.^36^ Nonetheless, p53 activity during reprogramming ensures elimination of cells bearing genomic aberrations. Therefore, disruption of p53 pathway increases the efficacy of reprogramming and the risk of mutations concomitantly.^37^,^38^ Down regulation of p53 activity has been shown to induce normal SCs transformation towards neoplastic, tumor cells.^39^ This may in turn result in cancer stem cells (CSs) formation. CSs share the fundamental properties of SCs, but their activity contributes to the cancer grow and maintenance instead of replenishing normal cell pool.^40^ Moreover, teratomas generated from p53 knockout iPSs showed the presence of double-strand DNA breaks and DDR activation, leading to the conclusion that p53 inhibition decreases genomic stability.^41^ Due to the high risk of tumor generation after transplantation, methods utilizing p53 inhibition in iPSs generation seem to be unsuitable for therapeutic use.

#### p53 in embryonic stem cells (ESCs)

It has been shown that p53 accumulates at low levels in the nucleus of hESCs, although in a deacetylated, inactive state. Apart from its canonical activity, p53 also performs a regulatory function over cell proliferation by controlling the expression of p21, known as cyclin-dependent kinase inhibitor. p21 inhibits the activity of cyclin/cdk2 complexes and restrains cell cycle progression. Dolezalova *et al.* revealed that after UVC-irradiation of hESCs, p21 mRNA is present, although its translation is inhibited by various microRNAs.^42^ However, a study by Maimets *et al.* contradicts these findings, revealing that the small molecule Nutlin, functioning as a p53 activator, elevates p21 protein levels in hESCs.^43^ Therefore, the role of p21 in the p53 pathway remains elusive.

p53 plays an important role in ESC differentiation. It has been shown that spontaneous differentiation occurs at significantly lower rates when the p53 level is reduced.^44^ However, one of the most crucial mechanisms supporting the theory of radiation-enhanced differentiation is the reduced expression of pluripotency factors driven by p53 activity. p53 binds directly to the promoters of NANOG and octamer-binding transcription factor 3/4 (Oct3/4), inhibiting their transcription. Moreover, elevated levels of p53 induce expression of differentiation markers GATA4 and GATA6.^43^ Furthermore, upon stabilization, in addition to its canonical function, p53 triggers the expression of miR-34a and miR-145, which subsequently repress the pluripotency factors Oct3/4, Kruppel-like factor 4 (Klf4), protein lin-28 homolog A (Lin-28A) and sex determining region Y-box 2 (Sox2), which supports differentiation.^45^

Retinoic acid (RA) is a commonly used differentiation factor utilized in various differentiation protocols, including those inducing the generation of neural cells, cardiomyocytes or chondrocytes.^46^–^48^ RA treatment results in the suppression of NANOG expression. However, this effect was not observed after p53 gene deletion, suggesting that p53 is required for RA-mediated NANOG suppression.^49^ Therefore, synergistic cooperation between these two proteins may be hypothesized.

It is important to mention that p53 also performs anti-differentiation stimulation through the Wnt canonical signaling pathway, which is responsible for the maintenance and self-renewal of human and murine ESCs.^50^,^51^

#### p53 in adult stem cells (ASCs)

Adult stem cells comprise endothelial progenitors cells (ESC) and hematopoietic stem cells (HSC) and tissue cells, called mesenchymal stem cells (MSC), found in many different organs of the human body and the one discussed in this review are listed in Table 1. Every ASC type contributes to a different cell lineage; therefore, any indications concerning radiation-enhanced differentiation may be true for some ASC types and completely false for others. To clarify the reasoning behind this statement, the properties of p53 activity in three different types of ASCs will be described: neural stem cells (NSCs), hematopoietic stem cells (HSCs) and mammary stem cells (MaSCs).

#### Neural stem cells (NSCs)

Neural stem cells have the potential to differentiate into neurons, astrocytes and oligodendrocytes. In adults, neurogenesis of the central nervous system begins within the subventricular zone (SVZ) and the subgranular zone of the dentate gyrus of the hippocampus, which serves as a niche for NSCs. The SVZ is a narrow zone of tissue in the wall of the lateral ventricle in the forebrain and is the most active neurogenic region in the adult brain.^52^ Neurons generated within SVZ migrate through a path called rostral migratory stream and reach their final destination within the olfactory bulb. A complete turn-over of resident cells within SVZ occurs every 2 to 4 weeks. Nearly 30 000 neuronal precursors are produced daily.^53^

It has been demonstrated that the neuronal progenitors of p53^−/−^ mice display a significantly higher proliferation rate compared to wild-type mice. NSCs can be maintained in culture as aggregates or neurospheres. p53^−/−^-derived NSCs formed substantially larger neurospheres than wild type cells, which was due to an increased number of cells per sphere, rather than larger cells. This finding indicates that one of the functions of p53 in NSCs is to restrain excessive proliferation.^54^

Monje *et al.* found that gamma irradiation of neural progenitor cells resulted in a higher efficiency of differentiation. Cultures irradiated with a 10.0 Gy dose showed increased differentiation compared to cells irradiated with a 2.0 Gy dose and control cells. However, the ratio between neurons and astrocytes/oligodendrocytes remained undisturbed, which is an important factor to consider in the context of radioenhancement.^55^

As previously mentioned, p53 also stimulates the Wnt signaling pathway. Data obtained by Wei *et al.* indicated that the Wnt/β-catenin signaling pathway plays a crucial role in the proliferation and differentiation of NSCs in the hippocampus. In this study, a low dose of ionizing radiation (0.3 Gy) was shown to activate the Wnt/β-catenin pathway. As a result, NSCs subjected to irradiation showed increased proliferation and differentiation with a concomitant decrease in apoptosis. Moreover, a water-maze test performed on mice indicated an improvement in the behavioral learning of these mice after low-dose irradiation compared to nonirradiated mice.^56^

#### Mammary stem cells (MaSCs)

Mammary stem cells are located in the mammary glands. They can differentiate into all lineages of mammary epithelial cells. MaSCs are also responsible for mammary gland development during puberty and pregnancy.^57^ MaSCs can be cultured *in vitro* as floating aggregates called mammospheres. A mammosphere is a spherical colony derived from a single MaSC by clonal proliferation.^58^ However, the division of MaSCs occurs predominantly by asymmetric division. Therefore, mammospheres usually contain a single stem cell surrounded by more differentiated progeny.^59^ Despite their self-renewal capabilities, they were shown to have a limited life span in culture conditions.

MaSCs derived from p53^−/−^ mice displayed an increased self-renewing potential, resulting in an increased number of MaSCs per mammosphere and an unlimited life span in culture conditions. This finding suggests that p53 is involved in the prevention of pathological proliferation by promoting asymmetric division, thus contributing to increased differentiation.^60^ It is also consistent with many scientific data regarding the role of p53 mutation in breast cancer development.^61^ Interestingly, MaSCs subjected to 4.0 Gy irradiation showed 2.7 fold increase in mammosphere reconstitution capacity, confirming that X-ray increases MaSCs proliferation.^62^

#### Hematopoietic stem cells (HSCs)

Hematopoietic stem cells (HSCs) are one of the best characterized human stem cells. For many years, they have been used in clinical applications, including leukemia treatment. HSCs differentiate into all of the blood cell lineages. They can be found in the red bone marrow. HSCs differentiate into myeloid and lymphoid progenitors, which may differentiate further giving rise to monocytes, erythrocytes, neutrophils and macrophages (myeloid progenitors) or T-lymphocytes, B-lymphocytes and NK-cells (lymphoid progenitors). The majority of HSCs reside in a quiescent state, while only a small fraction remains active and replenishes the blood cell pool.^63^

During steady-state hematopoiesis, p53 regulates HSC self-renewal and quiescence. It is also responsible for cell competition in the HSC niche. Cells expressing higher than average level of p53 undergo cell cycle arrest and senescence. This mechanism contributes to the maintenance of tissue homeostasis by the eradication of less functional cells.

Milyavsky *et al.* found that HSCs subjected to a 3.0 Gy irradiation dose exhibited a delayed DSB repair and an increased apoptotic response via the p53/antiphagocytic protein 1 (APP1) pathway compared to progenitor cells, which indicated a high sensitivity of HSCs to ionizing radiation.^64^ This finding is in agreement with the common notion that HSCs are one of the cell types most vulnerable to ionizing radiation (IR). However, despite its deteriorating effects, X-ray radiation has induced almost twofold increase in absolute number of murine HSCs. Increased number of murine HSCs in bone marrow was still detectable 2 months after irradiation. This effect was not observable in p21^−/−^ mice, suggesting p21 as a key factor of X-ray induced proliferation.^62^

### Mitochondria in the context of enhanced differentiation

Mitochondria are double-layered organelles that conduct the metabolic activities associated with energy production through oxidative phosphorylation. Their morphology varies between tissues and is strictly connected to the metabolic state of a given cell. In addition to tissue specific differences, mitochondria may undergo fusion or fission, giving rise to tubular or fragmented mitochondria, respectively. The fusion/fission mechanism is strictly connected with proliferation and differentiation.^65^ However, the outcome of tubular or fragmented mitochondria generation differs between cell types. In ESCs, the mitochondria reside in a fragmented state, and an increase in mitochondrial fusion precedes differentiation.

Ionizing radiation affects mitochondria in various ways. Mitochondrial DNA (mtDNA) is significantly more susceptible to IR compared to genomic DNA because it does not possess repair mechanisms as efficient as those found in the nucleus. Furthermore, mtDNA does not contain histones, which results in decreased resistance to various insults and a higher mutation rate.^66^ IR has also been found to induce both intracellular and mitochondrial oxidative stress.^67^ However, IR-induced mitochondrial production of reactive oxygen species (ROS) has been proven to be the most influential in mediating cellular damage compared to ROS generated in other compartments.^68^

Damage to mitochondria may trigger apoptosis, autophagy or, in the case of less severe lesions, fusion. This mechanism provides cross-complementation between impaired mitochondria, supporting their functionality by alleviation of IR-induced deficiencies.^69^ A 0.005 to 5.0 Gy dose of X-ray radiation has been shown to prompt a 1.5- to 3.8- fold increase in mitochondrial mass, which supports a theory of increased mitochondrial fusion after IR exposure.^70^

Lan *et al*. have shown that ESCs subjected to IR display a significantly increased level of ROS generation and metabolic activity.^25^ Both of these phenomena contribute to the induction of mitochondrial fusion, which in turn is a stimulus for differentiation. Therefore, it may be speculated that the radio-enhancement of differentiation could also involve changes in the mitochondrial fission/fusion machinery.

## Summary

**FIGURE 1.**
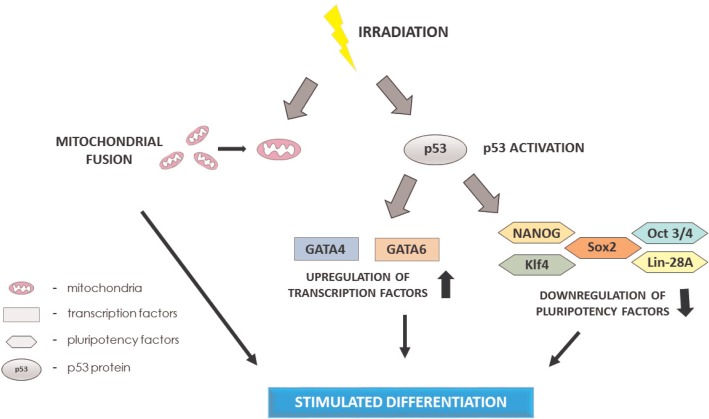
The pathway of radiation-enhanced differentiation.

A growing amount of evidence indicates that radiation-enhanced stem cell differentiation may become a potent tool for use in stem cell engineering (Figure 1). Ionizing radiation triggers an excessive amount of side effects and does not enable the use of directed differentiation as a sole method in stem cell applications. However, a proper dosage may increase its efficacy while concomitantly reducing its disadvantages. It is important to highlight that despite very efficient mechanisms of DDR in the majority of stem cells, IR bears the risk of introducing genomic instability. Therefore, it is of great importance to define the radiation dose that maximizes the stimulation of differentiation and minimizes the genotoxic effects. The response to irradiation varies between different stem cell types; thus, each type of stem cell requires an independent evaluation of dosage. The deteriorating effects of irradiation could also be partially overcome by the formation of embryoid bodies, which display a significant increase in radioresistance compared to human embryonic stem cells (hESCs). It is also important to note that there are significant discrepancies between murine and human cell models in response to IR (Table 2.); thus, any assumptions based on murine models should be confirmed in human cells.^71^ Nonetheless, despite the presence of molecular evidence indicating the probable application to stem cell differentiation methodologies, the concept of radiation-enhanced stem cell differentiation remains to be scientifically proven.

## Figures and Tables

**TABLE 1.** The examples of adult stem cell (ASC) types and their corresponding tissue of origin, progenitors and fully differentiated cells

**Organ**	**Stem cell type**	**Progenitors and fully differentiated cells**
Bone marrow	Hematopoietic stem cells	Myeloid progenitor cells, Lymphoid progenitor cells
Intestine	Intestinal stem cells	Enterocytes, Goblet cells, Entero-endocrine cells, Paneth cells
Brain	Neural stem cells	Neurons, Astrocytes, Oligodendrocytes
Mammary gland	Mammary stem cells	Luminal cells, Myoepithelial cells
Muscle	Myosatellite cells	Mioblasts

**TABLE 2.** Examples of differences between human and murine cells affecting IR response

**Differences between human and murine DNA repair mechanisms**	**References**
Murine cells are deficient in p53 global genomic repair	72
Human ESC rejoin X-ray induced DSB faster than murine ESC	71
Murine cells repair DNA base damage more efficiently	73
Murine cells are more sensitive to oxidative stress	74,75
Murine cells are more prone to oncogenic transformation	76,77

DSB = double strand breaks; ESC = embryonic stem cells
